# Understanding and modifying starch metabolism to limit yield losses in field-grown cassava

**DOI:** 10.1093/plphys/kiag341

**Published:** 2026-06-04

**Authors:** Laure C David, Gabriel Deslandes-Hérold, Carmen Hostettler, Sylvain Bischof, Michaela Fischer-Stettler, Anna V Carluccio, Barbara Pfister, Gavin M George, Wuyan Wang, Livia Stavolone, Andreas Gisel, Simon E Bull, Melanie R Abt, Samuel C Zeeman

**Affiliations:** Plant Biochemistry, Institute of Molecular Plant Biology, ETH Zurich, Zurich 8093, Switzerland; Plant Biochemistry, Institute of Molecular Plant Biology, ETH Zurich, Zurich 8093, Switzerland; Plant Biochemistry, Institute of Molecular Plant Biology, ETH Zurich, Zurich 8093, Switzerland; Plant Biochemistry, Institute of Molecular Plant Biology, ETH Zurich, Zurich 8093, Switzerland; Plant Biochemistry, Institute of Molecular Plant Biology, ETH Zurich, Zurich 8093, Switzerland; Istitute for Sustainable Plant Protection, Department of Bio-Agrifood Sciences, National Research Council (CNR), Bari, Italy; International Institute of Tropical Agriculture, Ibadan, Oyo State, Nigeria; Plant Biochemistry, Institute of Molecular Plant Biology, ETH Zurich, Zurich 8093, Switzerland; Plant Biochemistry, Institute of Molecular Plant Biology, ETH Zurich, Zurich 8093, Switzerland; Plant Biochemistry, Institute of Molecular Plant Biology, ETH Zurich, Zurich 8093, Switzerland; Istitute for Sustainable Plant Protection, Department of Bio-Agrifood Sciences, National Research Council (CNR), Bari, Italy; International Institute of Tropical Agriculture, Ibadan, Oyo State, Nigeria; International Institute of Tropical Agriculture, Ibadan, Oyo State, Nigeria; Institute for Biomedical Technologies, National Research Council (CNR), Bari, Italy; Plant Biochemistry, Institute of Molecular Plant Biology, ETH Zurich, Zurich 8093, Switzerland; Plant Biochemistry, Institute of Molecular Plant Biology, ETH Zurich, Zurich 8093, Switzerland; Plant Biochemistry, Institute of Molecular Plant Biology, ETH Zurich, Zurich 8093, Switzerland

## Abstract

The carbohydrate-rich storage roots of cassava (*Manihot esculenta*) are among the world's most vital staple foods, providing food security and income for hundreds of millions of people—primarily small-holder farmers in tropical and subtropical regions. Cassava is also a major source of starch for both food and industrial applications. Root yield is largely determined by starch content, which acts as the main sink for photoassimilates during vegetative growth. In addition, stored starch serves as a carbohydrate reservoir that supports regrowth after stress events, such as drought or shoot pruning, or during stem propagation. To investigate source-sink dynamics and identify key factors in sink metabolism, transcriptomic and proteomic analyses of cassava storage roots were conducted following shoot pruning. This perturbation led to a significant reduction in root starch content and triggered widespread transcriptional reprogramming, restricting respiration and growth. Notably, key starch biosynthesis genes were repressed, while starch degradation genes—including the plastidial *α-AMYLASE3 A* (*AMY3A*)—were induced. To confirm the role of AMY3 in root starch breakdown, *AMY3A*-suppressed cassava lines were generated via RNA interference and evaluated under both greenhouse and field conditions. These lines exhibited up to a 7.5-fold reduction in starch mobilization following pruning compared to controls, demonstrating AMY3's key function in storage root starch degradation—a role that contrasts with its redundancy in systems like transitory starch degradation in *Arabidopsis* leaves. Crucially, *AMY3A* suppression did not impair cassava stem cutting regrowth, highlighting it as a promising target for improving cassava root traits and advancing food security.

## Teaser

Suppressing the starch-degrading enzyme AMY3A in cassava reveals its key role in storage root starch breakdown and offers a promising strategy to limit yield losses in the field.

## Introduction

In sub-Saharan African countries, approximately one-third of the population relies on cassava (*Manihot esculenta* Crantz) for most of their calorific intake (Szyniszewska 2020), ranking it among the most important food crops globally. The starch-rich storage roots are also processed, especially in Southeast Asia, for use in the manufacturing of biodegradable plastics and packaging materials, paper, medical products, and food additives. Cassava is therefore abundant in everyday life, supporting small-holder farmers in producing food for personal consumption as well as contributing to local, regional, and international value chains. Despite its global importance, cassava remains poorly studied compared with other staple crops.

Cassava storage roots are major carbon sinks, providing exceptional quantities of nonstructural carbohydrates (NSCs; especially starch and some soluble sugars) that make up 70% to 90% of the root dry weight. Early tracer experiments using ^14^CO_2_ fed to leaves revealed that, during rapid growth of storage roots, 60% of the label was detected in the root after 7 d (Hume 1975). The starch-rich roots can be harvested as a crop. Under certain conditions, however, they can also serve as an energy reserve for the plant. In such cases, a sink-to-source inversion occurs, and stored carbohydrates are remobilized to support respiration and canopy regrowth (e.g. with the first rains after the dry season (Laya et al. 2018), or after the plant is pruned). Such remobilization of starch leads to a reduction in root dry matter and crop quality (van Oirschot et al. 2000; Ayoola and Agboola 2004). Nevertheless, pruning is performed by farmers for various reasons, including use of leaf material as a vegetable or livestock feed, for intercropping practices, for stem multiplication, or to prevent damage from inclement weather. Importantly, shoot pruning prior to harvest also serves to minimize subsequent post-harvest physiological deterioration—the consequence of oxidative damage that can render freshly harvested roots unpalatable within approximately 72 h (Wheatley and Schwabe 1985; Mehdi et al. 2019). Improved understanding of the genetic reprogramming that occurs in roots after shoot pruning will help to identify factors linked to starch catabolism and/or crop deterioration, which is therefore important research foci that can aid the delivery of trait-enhanced crops for improved food security (Chiewchankaset et al. 2019; Mehdi et al. 2019; Obata et al. 2020).

Starch forms as semi-crystalline, insoluble granules inside plant cell plastids (chloroplasts in leaves and amyloplasts in non-green tissues) and is composed of the two glucose polymers, amylopectin and amylose (Smith and Zeeman 2020). Amylopectin is a large molecule (10^7^ to 10^9^ Da) comprised of α-1,4-linked chains branched via α-1,6-linkages, resulting from the combined actions of starch synthases, branching enzymes, and debranching enzymes (Pfister et al. 2014; Pfister and Zeeman 2016). The architecture of amylopectin enables it to form a layered, semi-crystalline matrix. In contrast, amylose is smaller (10^4^ to 10^6^ Da) and comprised primarily of linear α-1,4-linked chains generated by a single enzyme, granule-bound starch synthase embedded within the amylopectin matrix (Seung 2020).

The key steps in starch degradation differ, depending on the plant species and organ type, but the exact mechanism in cassava roots is not known. In Arabidopsis leaf chloroplasts, the first step is the reversible phosphorylation of the starch granule surface, which disrupts the semi-crystalline structure, allowing hydrolases—chiefly β-amylases and debranching enzymes (Ritte et al. 2002; Kötting et al. 2005; Edner et al. 2007)—to act. A similar system likely operates in the amyloplasts of potato tubers (Ritte et al. 2002). In contrast, in the endosperm of germinated cereal seeds, starch degradation in the non-living starchy endosperm is initiated by α-amylases secreted from surrounding living cells. The α-amylases hydrolyze internal α-1,4 linkages, acting at specific sites (pores) on the granule surface without the involvement of reversible glucan phosphorylation (Sun and Henson 1990; Blennow et al. 2000).

Plant α-amylases (AMY) can be categorized into three distinct classes based on their amino acid sequences (Stanley et al. 2005; Ju et al. 2019). The AMY1-class proteins are typically targeted to the secretory pathway and include those involved in starch degradation in the cereal endosperm (e.g. *AmyI*-1 in rice; Asatsuma et al. 2005). The AMY2-class proteins are localized in the cytosol and have been implicated in starch degradation and malto-oligosaccharide metabolism during potato storage (cold-induced sweetening) and sprouting (*StAMY23*; Zhang et al. 2014; Hou et al. 2017). AMY2-class proteins are also implicated in starch-to-sugar conversion during the curing of harvested tobacco leaves (*NtAMY1*-1; Yamaguchi et al. 2013). The AMY3-class proteins are targeted to the plastids and contain N-terminal extensions with two carbohydrate-binding modules that facilitate substrate interactions (Glaring et al. 2011; Seung et al. 2013). Although AMY3 is present in Arabidopsis leaf chloroplasts, it is not essential for starch degradation (Yu et al. 2005) due to redundancy with other enzyme classes, i.e. β-amylases and debranching enzymes. Induction of the AMY3-class has been observed during starch degradation in crops (e.g. in ripening banana fruit; Xiao et al. 2018), but they have not been shown to be required for starch degradation to occur.

Here, we identify a number of highly-induced proteins in the cassava storage root after shoot pruning to induce a sink-source inversion, which represent targets to limit yield losses. A putative plastidial α-amylase, *α-AMYLASE3A* (*AMY3A*), is amongst these targets. We show that *AMY3A* encodes an active enzyme, confirm its plastidial localization and demonstrate that its suppression using RNAi reduces post-pruning mobilization of root starch under field conditions. This highlights starch degradation in general and *AMY3A* in particular as targets for cassava crop improvement.

## Results

### Carbohydrate levels and fluxes in cassava

**Figure 1:**
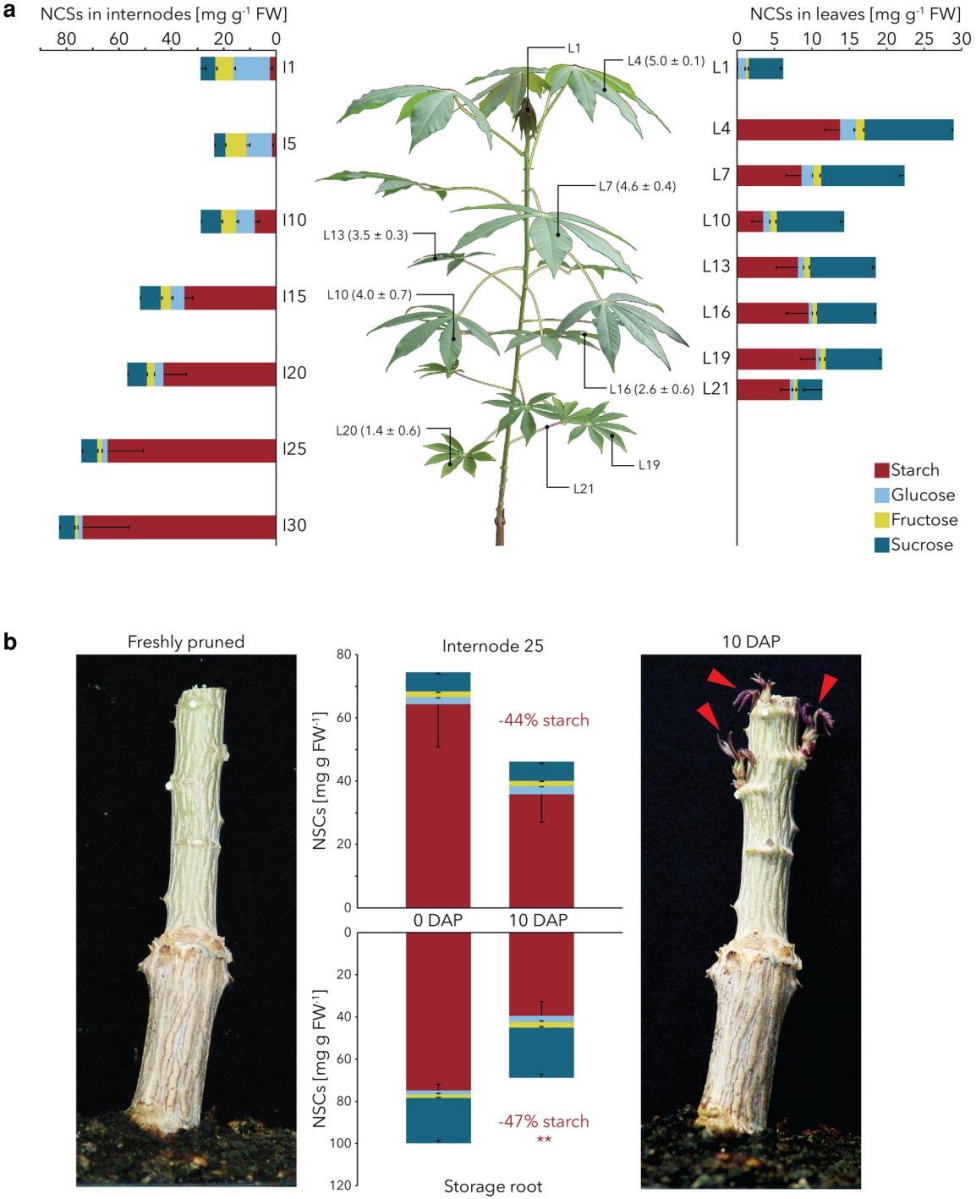
Photosynthesis and nonstructural carbohydrates in 5-month-old cassava plants and resource mobilization after shoot pruning. a) Internodes (I) and leaves (L), numbered from top to bottom; L1, the first unfurled leaf; L4, the first fully expanded leaf. I1 is between L1 and L2. Center: mean (± SE) photosynthesis rates are provided in parentheses for indicated leaves (as µmol CO_2_ m^−2^ s^−1^ at 100 µmol photons m^−2^ s^−1^). Data are from three biological replicate plants. In each case, duplicate measurements were made at each of 2 to 3 sites. Right: measurements of the indicated NSCs in hexose equivalents in selected leaves (*n* = 2 to 5 biological replicates). Left: NSCs in selected internodes (*n* = 3 to 5 biological replicates). Means ± SE provided. FW, fresh weight. See also Dataset S1, a and b. b) Plants were pruned and storage roots harvested immediately or 10 DAP. Within this period, shoots regrew (arrowheads). NSCs were measured at both timepoints in the stem (I25, same data as in a); *n* = 3 to 5 biological replicates) and in storage roots (*n* = 3 to 5 biological replicates). Means ± SE provided. The starch content in the stem and the storage root decreased upon pruning (highly significant in the root; Student's *t*-test, **, *P* < 0.01). See also Dataset S1c.

To improve our understanding of source-sink carbon allocation in cassava, NSCs were measured in leaves and stem internodes of 5-month-old glasshouse-cultivated plants (Dataset S1). The youngest unfurled leaf, designated leaf 1, contained sucrose but lacked starch, suggesting it is a sink for sugars translocated from older leaves (Fig. 1a). Feeding ^14^CO_2_ to leaf 8 to track photoassimilates confirmed export to leaves 1 to 3. No ^14^C was detected in leaf 4, suggesting that it had transitioned to a source leaf (Figure S1). Consistent with this, leaf 4 had high photosynthetic rates and substantial amounts of both sucrose and starch (Fig. 1a). Older leaves had less NSCs and progressively lower photosynthetic capacity (e.g. leaf 20 had 30% the capacity of leaf 4; Fig. 1a). Older leaves also did not import ^14^C from leaf 8, despite their low photosynthetic capacity. However, basipetal phloem transport was evident from the appearance of ^14^C in the phloem parenchyma in the exterior of the developing storage roots (Figure S1; Mehdi et al. 2019).

The stem sucrose content was similar between internodes, but the levels of other carbohydrates differed markedly (Fig. 1a); the younger internodes had low starch content, but higher quantities of glucose and fructose, presumably derived from sucrose hydrolysis. In contrast, starch content increased markedly with stem age, showing that it is an important sink for photoassimilates, while hexose levels declined.

### Shoot pruning triggers storage root starch degradation and transcriptional reprogramming

To perturb the source-sink balance between shoot and root, glasshouse-cultivated cassava stems were pruned (ratooned) with 10 cm above the soil remaining attached to the root system. Ten days after pruning (DAP), axillary buds emerged along the stem and starch content had decreased in stems (immediately below the pruning site) and storage roots, compared with the time of pruning (Fig. 1b). Starch mobilization was particularly marked in the storage root, where starch content decreased by 47% at 10 DAP. However, no significant changes in sucrose or hexose content were observed. This suggests that the onset of starch degradation supported respiration and maintained sugar levels to initiate shoot regrowth, presumably with the conversion of sink tissues to source tissues. In the stem, the average starch content was 44% lower after pruning but this was not statistically significant (*P* = 0.11).

**Figure 2:**
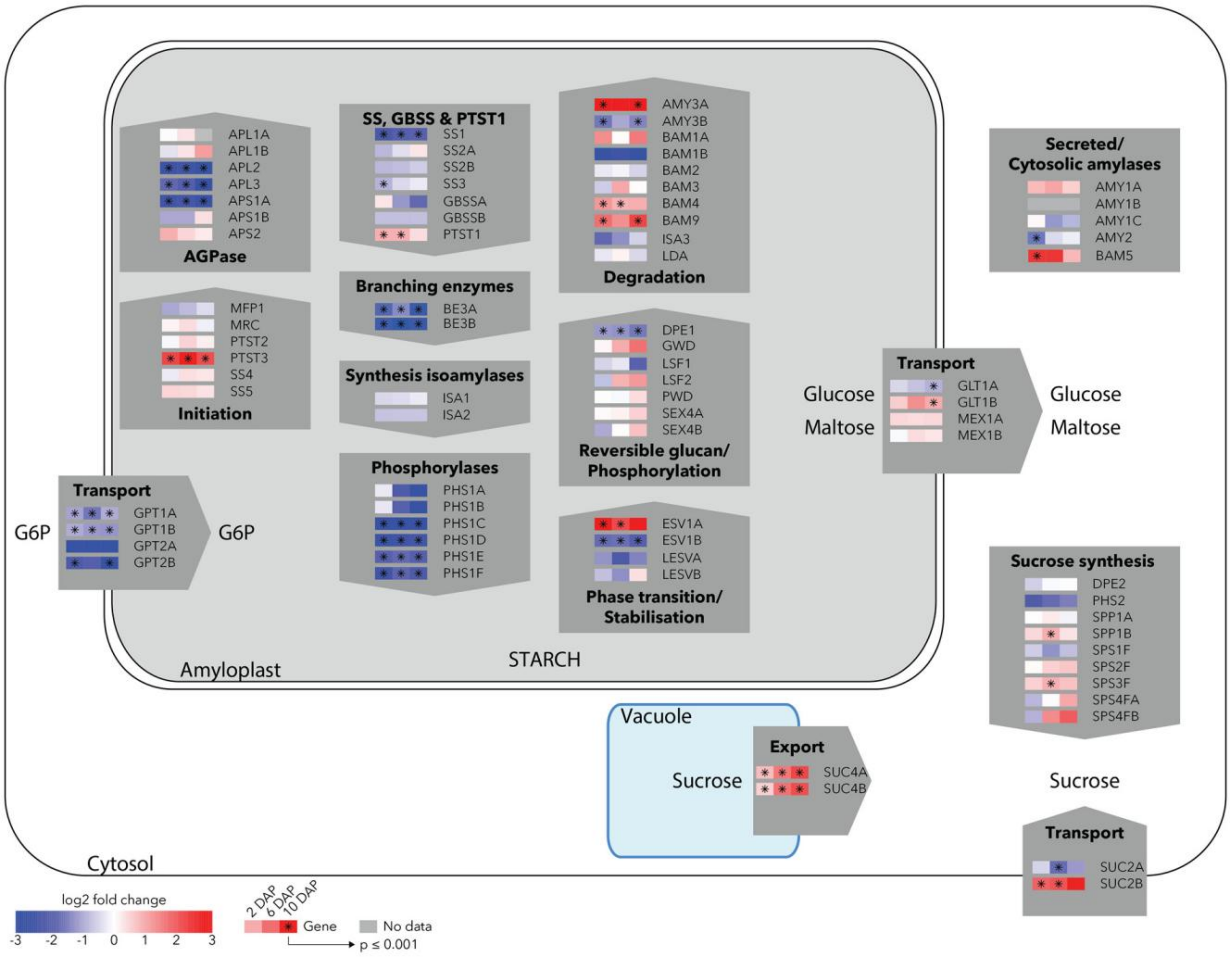
Change in expression of starch-metabolism related genes in storage roots upon shoot pruning. Normalized RNA-seq expression values from storage roots of unpruned plants harvested at the same time on two consecutive days (day 0 and day 1) were used as controls to calculate the log_2_ fold change in the designated transcripts at 2, 6, and 10 DAP. G6P, glucose 6-phosphate. Boxes highlighted with a star are highly significant changes relative to the control samples (Student's *t*-test; *P*-values ≤0.001). See Figure S2 and Dataset S2 for quality control, underlying data, gene annotations, and statistical analysis.

To identify the genes involved in the change of the storage root from a sink into a source tissue, particularly those affecting the large change in starch content, transcriptomic profiling was performed on storage roots harvested from pruned plants over a time-course starting 4 h after pruning (HAP) and subsequent sampling at 1, 2, 6, and 10 DAP (Dataset S2). Control samples were harvested from unpruned plants at time 0, after 4 h, and after 1 d (Figure S2a). A sample-to-sample distance matrix of the transcriptomes (consisting of 10,775 reliably detected transcripts) revealed that pruned and control samples from the 4 HAP and 1 DAP time points all clustered together (Figure S2b). This suggests that major changes in storage root gene expression occurred only after more than 1 d. The transcriptional changes formed five clusters, and subsequent enrichment analysis for gene ontology (GO) terms revealed downregulation of genes associated with glycolysis, the tricarboxylic acid cycle and oxidative phosphorylation, as well as genes associated with protein biosynthesis and cell division (Figure S3). This suggests that loss of sucrose delivery to the root due to pruning triggers a restriction in root respiration and growth. Although many genes were upregulated, the GO enrichment analysis did not reveal clear patterns (Dataset S2d). We focused on starch metabolic genes whose expression changed in samples harvested from 2 DAP onwards (Fig. 2). At these time points, several genes encoding proteins involved in starch synthesis were downregulated in the roots of pruned plants. These included genes encoding glucose 6-phosphate transporters of the plastid envelope, subunits of the ADP-glucose pyrophosphorylase (the regulated step of starch biosynthesis), as well as isoforms of starch synthases and branching enzymes (Fig. 2). Interestingly, α-glucan phosphorylase was also substantially downregulated, consistent with recent work implicating it in the process of starch granule initiation (Seung et al. 2025). In contrast, a number of genes involved in starch degradation were upregulated—in particular, an orthologue of the Arabidopsis plastidial α-amylase gene, *AMY3* (Seung et al. 2013), which was one of the most strongly upregulated genes. Interestingly, the orthologue of Arabidopsis β-amylase 9 gene (*BAM9*) was also strongly upregulated. This is conspicuous since, in Arabidopsis, BAM9 is a non-enzymatic positive regulator of starch degradation (David et al. 2022). Together, these results indicate that storage root starch biosynthesis is halted and its degradation induced upon shoot pruning, changes possibly triggered by a cessation of incoming sucrose. Interestingly, the gene encoding KINγ, a subunit of the SNF1-related protein kinase, known to be a major regulator of carbon allocation (Jamsheer et al. 2021) was also highly upregulated. Finally, transcripts of both gene orthologs of the Arabidopsis vacuolar sucrose exporter 4 (*SUC4*) were increased, suggesting a shift from carbon storage to its utilization (Schneider et al. 2012), as was a homolog of the *SUC2* gene, potentially involved in active transport of sucrose from the storage root.

We complemented our transcriptome analysis using shotgun proteomics of storage root samples collected before pruning and 10 DAP (Dataset S2). Although fewer proteins than transcripts were detected, correlative analyses revealed 120 candidate genes that showed a robust response, with significant changes in both transcript levels at 2 DAP and protein content at 10 DAP; Figure S4). This included a downregulated expression of a subunit of ADP-glucose pyrophosphorylase, APL3, as well as the induced expression of AMY3 and KINγ proteins. GO enrichment analysis revealed downregulation of proteins associated with cellulose biosynthesis, Golgi function and cytokinesis, all consistent with a restriction in root growth (Dataset S2e).

### AMY3A is a plastid-targeted α-amylase

**Figure 3:**
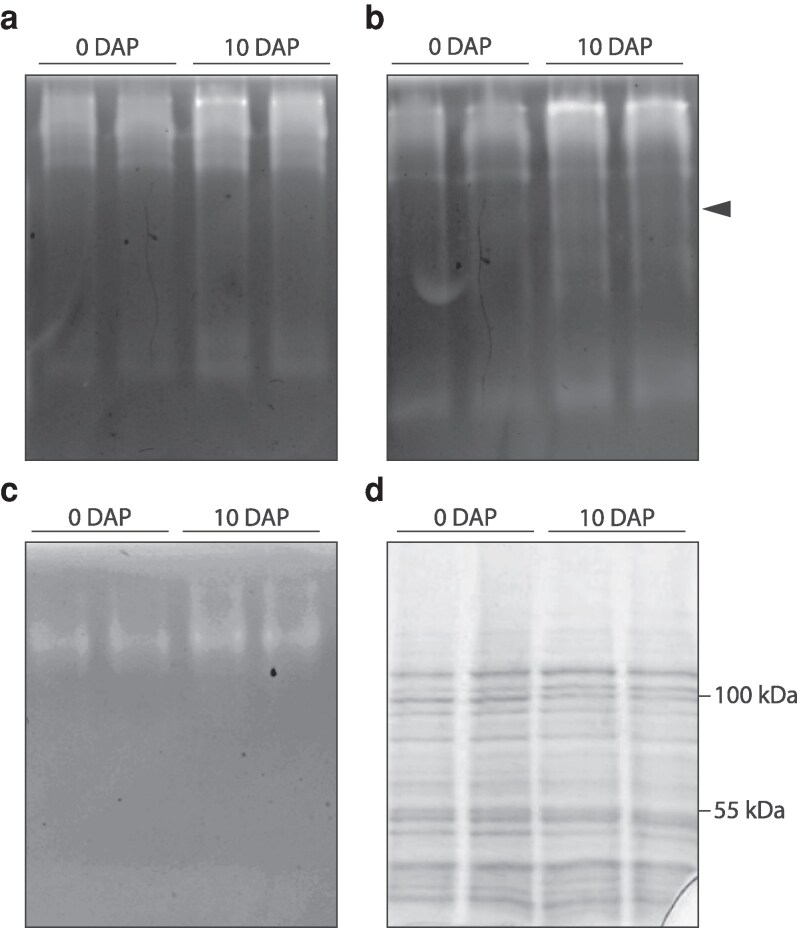
Zymograms of starch-metabolizing enzymes. Duplicate crude extracts from storage root harvested 0 and 10 DAP were subjected to native PAGE containing different substrates for amylolytic enzymes. a) 0.1% (w/v) potato amylopectin, 2.5 µg protein was loaded. b) 0.1% (w/v) β-limit dextrin, 2.5 µg protein was loaded. The arrowhead indicates the anticipated location of AMY3, based on prior work in Arabidopsis. c) 1% (w/v) Red Pullulan, 7.5 µg protein was loaded. d) Coomassie staining to confirm equal protein loading (7.5 µg were loaded).

We decided to focus on the induction of starch degradation and test whether modulating this induction could restrict the mobilization of storage root reserves. To investigate the amylolytic activities in storage roots upon pruning, zymograms utilizing different glucan substrates were performed. On gels containing amylopectin (a substrate for most amylolytic enzymes, including α-amylases, BAMs, isoamylase- (ISA), and limit dextrinase- (LDA)-type debranching enzymes), protein extracts from storage roots of pruned plants had increased amylolytic activities compared to extracts of unpruned plants (Fig. 3a). Increased activity was also seen on gels containing β-limit dextrin (a substrate resistant to BAMs, but susceptible to AMY, ISA, and LDA activities; Fig. 3b). It is not possible to ascribe these activities to specific cassava enzymes. However, based on previous work in Arabidopsis, the upper-most bands likely contain multiple co-migrating activities including isoamylase and β-amylase isoforms (Zeeman et al. 1998a; Schreier et al. 2019). In Arabidopsis, AMY3 has a slightly higher mobility (Yu et al. 2005), and a faint activity in this area of the β-limit dextrin gels was detected in extracts from storage roots of pruned plants (indicated by the arrowhead in Fig. 3b). Increased activity was seen on gels containing Red Pullulan, a dyed substrate specific to LDA (Fig. 3c). Coomassie staining of the protein samples separated by SDS-PAGE confirmed similar protein loading (Fig. 3d).

**Figure 4:**
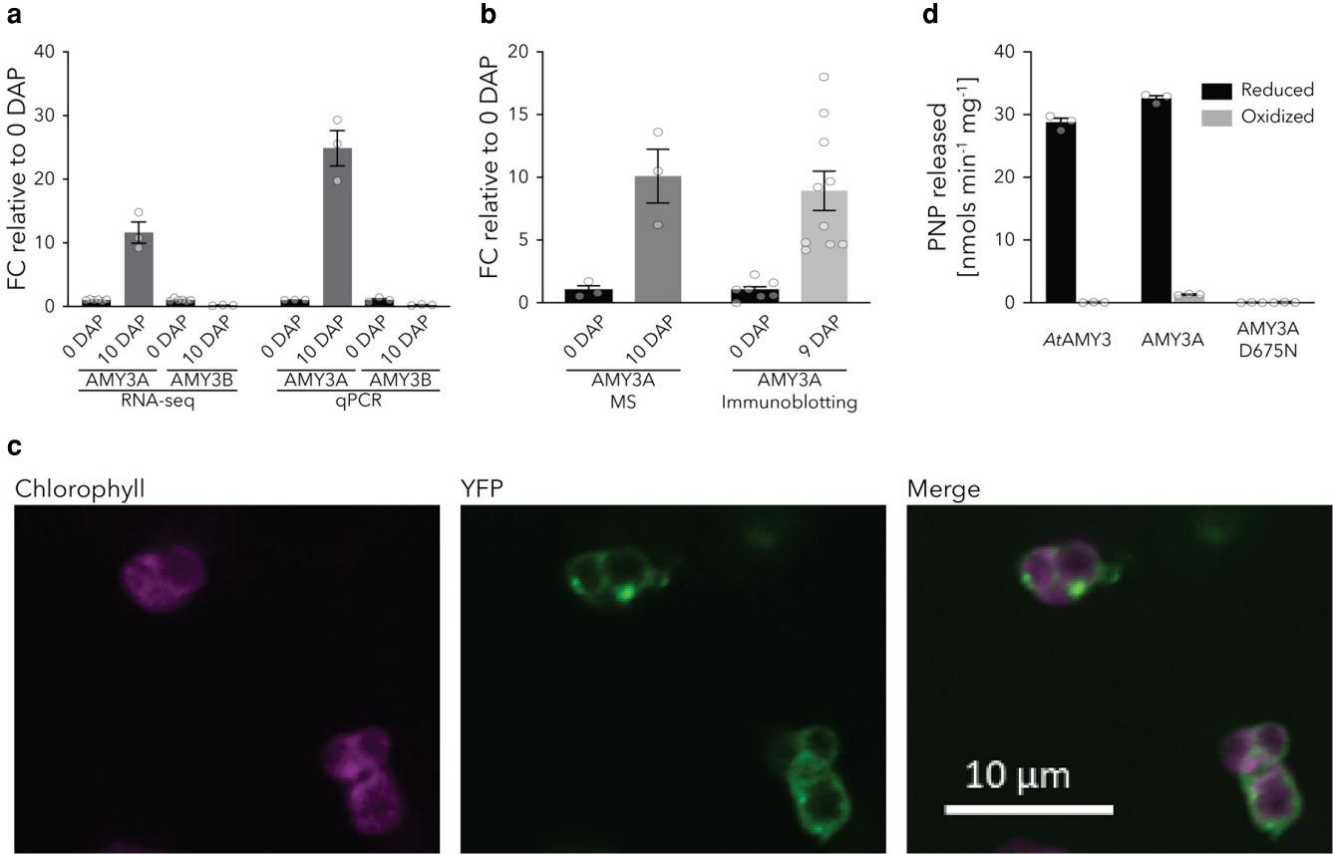
Validation of the accumulation of AMY3A transcripts and proteins in storage roots upon pruning. a) Induction of AMY3A and AMY3B gene expression in storage roots in response to pruning. Shown are RNA-sequencing and RT-qPCR-derived data at 0 (bars) and 10 DAP. For RNA-sequencing, the normalized expression values from Manes.05G097100 (*MeAMY3A*) or Manes.01G189800 (*MeAMY3B*) were divided by the average of the normalized expression values of housekeeping genes: Manes.15G054700 (*MeEF1aA*), Manes.15G054800 (*MeEF1aB*), Manes.15G054900 (*MeEF1aC*), and Manes.04G111000 (*MeUBQ10*). For each sample, the FC was then calculated by dividing with the average values at 0 DAP. *n* = 3. Shown are means ± SE. b) Level of AMY3A protein in storage roots before and after pruning. Shown are MS and immunoblot-derived data at 0 DAP (black bars) and 10 or 9 DAP, respectively. *n* = 3 for MS samples and *n* = 7 to 10 for immunoblotting experiments. Shown are means ± SE. c) Subcellular localization of AMY3A-YFP transiently expressed in *Nicotiana benthamiana* leaves. d) Activity of recombinant *At*AMY3, AMY3A, and AMY3A D675N enzymes using BPNP-G7 as a substrate under reducing or oxidizing conditions. *n* = 3. Shown are means ± SE. See Dataset S3.

The increased amylolytic activities and the strong induction of an *AMY3* gene led us to focus on the role of this putative plastidial α-amylase. Of the six cassava α-amylase genes (Yang et al. 2023), three are orthologous to *AtAMY1*, one to *AtAMY2*, and two to *AtAMY3* (hereafter named *AMY3A* and *AMY3B*; Figure S5). Based on RT-qPCR analysis, *AMY3A* but not *AMY3B* expression was increased at 10 DAP (Fig. 4a; Dataset S3). Further, *AMY3A* expression was approximately 120-fold higher than *AMY3B*. The increased AMY3A protein observed via proteomics was corroborated by immunoblotting using polyclonal antibodies raised against recombinant *Me*AMY3A (Fig. 4b).

TargetP 2.0 predicted both AMY3A and AMY3B to have chloroplast transit peptides (Figure S5). Transient expression of the AMY3A-YFP in *Nicotiana benthamiana* leaves revealed that the YFP signal overlapped with the chlorophyll signal, confirming that the protein localizes to plastids (Fig. 4c). Biochemical analysis of recombinant AMY3A confirmed redox-dependent amylolytic activity toward blocked *P*-nitrophenyl maltoheptaoside (BPNP-G7; an artificial substrate specific for endoamylases; Fig. 4d; Dataset S3). Furthermore, mutation of the putative active site (D675N) disrupted its catalytic activity, together suggesting MeAMY3 to be similar to *At*AMY3, the redox-activated α-amylase of Arabidopsis (Seung et al. 2013).

### AMY3A suppression reduces starch mobilization upon pruning

**Figure 5:**
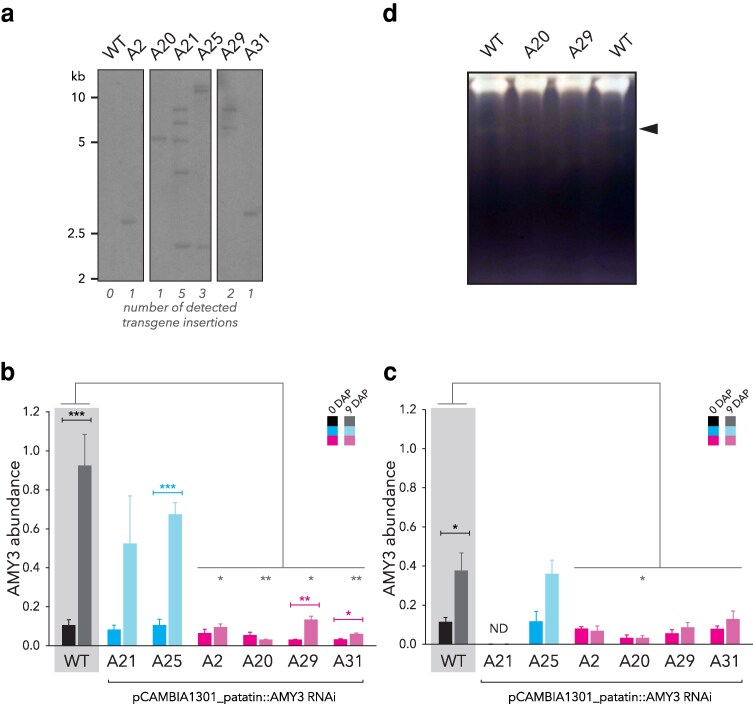
AMY3A RNAi lines: insertion numbers and AMY3 repression. a) Southern blot was performed on HindIII-digested genomic DNA. ^32^P-labeled hygromycin probes were used to detect the core sequence of the transgene cassette, which contains no HindIII restriction sites. The detected number of transgene insertions is indicated below. b) Relative quantification of AMY3 protein in roots. c) Relative quantification of AMY3 protein in stems. In b) and c), AMY3 abundance was determined by immunoblot analysis of protein extracts of glasshouse-grown WT and transgenic line samples at 0 and 9 DAP. The ratio of fluorescence of the AMY3 signal relative to the actin signal was calculated and normalized to an internal control (a WT root sample) present on every blot. AMY3 levels were compared within each line between 0 and 9 DAP as well as within timepoints, in this case, with respect to the WT (as indicated). ND, not determined. *n* = 3 to 10; shown are means ± SE. Note that the data for the WT displayed in b) is the same as in Fig. 4b, but normalized differently. Asterisks indicate statistically significant differences and are based on unpaired *t*-tests. *, *P* ≤ 0.05; **, *P* ≤ 0.01, ***, *P* ≤ 0.001. For b) and c), see also Dataset S4. d) Zymogram analysis of crude extracts from storage roots harvested 10 DAP using native polyacrylamide gels containing 0.2% (w/v) β-limit dextrin (2.5 µg proteins loaded). The arrowhead indicates the putative AMY3 activity.

To assess the influence of AMY3A on starch degradation, we selected six transgenic lines carrying an RNAi construct for storage root-specific *AMY3A* suppression. These lines represented independent transgenesis events (single-locus insertions in lines A2, A20, and A31, and multi-locus insertions in lines A21, A25, and A29; Fig. 5a). AMY3 protein accumulation in storage roots was suppressed in A2, A20, A29, and A31 after pruning of glasshouse-cultivated plants, indicating the RNAi construct was functional in these lines (Fig. 5b; Dataset S4). Suppression was incomplete in line A21, and no suppression was observed in line A25, and these lines were subsequently used as transformation controls. The high numbers of T-DNA insertions in these lines (5 and 3, respectively) may have triggered transcriptional gene silencing, causing weak siRNA production. Given that cassava stems also contain starch (Fig. 1), we assessed AMY3 levels in this tissue. Indeed, AMY3 was also induced in stems in response to pruning, although to a lesser extent than in storage roots (Fig. 5c). *AMY3A* induction was also suppressed in the RNAi lines’ stems, consistent with a recent report of some patatin B33 promoter activity in cassava stem tissues (Zierer et al. 2022). We repeated the zymogram analysis on proteins extracted from storage roots of two of the transgenic lines (A20 and A29). Using a higher concentration of β-limit dextrin, the faint band of activity tentatively ascribed to AMY3 (Fig. 3b) could be detected in extracts of the wild-type (WT) but not the RNAi lines (Fig. 5d).

**Figure 6:**
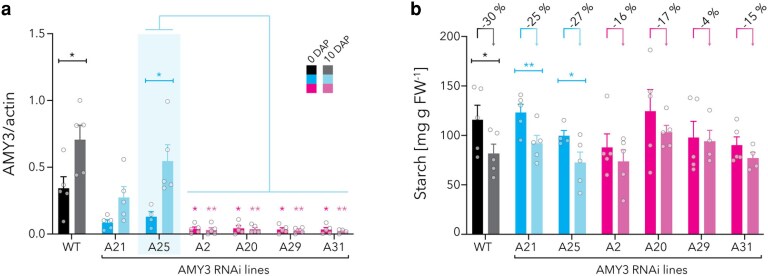
AMY3 expression and starch degradation in storage roots upon pruning in field-grown RNAi lines. a) Relative quantification of AMY3 protein in storage roots of WT and transgenic lines at 0 and 10 DAP. AMY3 abundance was determined as the ratio of fluorescence of AMY3 signal to actin signal. For each sample, the calculated ratio was normalized to an internal control present on every blot. *n* = 4 to 5; shown are means ± SE. AMY3 levels were compared within each line between 0 DAP and 10 DAP as well as within timepoints, in this case with respect to reference line A25 (highlighted background, as indicated). Asterisks indicate levels of statistical significance (*, *P* < 0.05; **, *P* < 0.01) and are based on unpaired *t*-tests. For statistical analysis against the WT, see Dataset S5a. b) Starch content in storage roots of WT and transgenic lines at 0 and 10 DAP. *n* = 4 to 5; shown are means ± SE. Starch contents were compared within each line between 0 DAP and 10 DAP. Asterisks indicate levels of statistical significance (*, *P* < 0.1; **, *P* < 0.05) and are based on unpaired *t*-tests. See also Dataset S5b.

The *AMY3A* RNAi lines were grown in a confined field site at the International Institute for Tropical Agriculture (Ibadan, Nigeria) and assessed after 6 mo. As in glasshouse-grown plants, AMY3 protein was significantly increased in harvested wild-type storage roots 10 DAP, as well as in control line A25, but not in lines A2, A20, A29, and A31 (Fig. 6a; Dataset S5). Line A21 had intermediate AMY3 levels. Also consistent with earlier observations (Fig. 1b), the starch content of the storage roots of wild-type plants dropped markedly (by 30%) by 10 DAP (Fig. 6b). Comparable mobilization of starch reserves was observed in the transgenic control lines A21 (−25% starch) and A25 (−27% starch). However, in the transgenic lines with suppressed AMY3, the reductions in starch yield were lower in the roots of pruned plants (16%, 17%, 4%, and 15% in lines A2, A20, A29, and A31, respectively; Fig. 6b).

Under laboratory conditions, Arabidopsis plants lacking AMY3 had normal transitory starch metabolism in leaves and no negative effects on vegetative growth were reported (Yu et al. 2005). To assess this in cassava, we characterized the *AMY3A* RNAi plants with regard to their general growth in the protected field site in Nigeria and assessed agronomically relevant parameters without and with pruning (Figure S6). The processes associated with transgenesis in tissue culture can result in genetic/epigenetic effects causing somaclonal variation in agronomic traits after regeneration (Kitimu et al. 2015; Beyene et al. 2016). Therefore, in addition to the wild-type, we compared lines with clear repression of AMY3 (A2, A20, A29, and A31) to line A25, which had undergone all transformation and selection processes, but is wild-type-like in relation to AMY3 induction (Fig. 5b; Fig. 6a) and root starch mobilization (Fig. 6b). The shoot and storage root fresh weights of all transgenic lines were comparable, as were the harvest indexes, suggesting that the repression of AMY3 per se did not take a toll on plant biomass allocation. However, the harvest index of the wild-type was higher than all of the transgenic lines (Table 1; Dataset S6).

**Table 1:** Agronomic performance of field-grown AMY3 RNAi lines.

Plant line	Shoot FW [kg plant^−1^] *n* = 5	Storage root FW [kg plant^−1^] *n* = 5	Harvest index *n* = 5	Percentage of nodes resprouting from rootstocks at 10 DAP *n* = 5	Percentage of nodes resprouting from directly planted stem cuttings after 40 d *n* = 8 to 10	Percentage of nodes resprouting from stored stem cuttings after 30 d *n* = 8 to 10
**WT**	4.49 ± 0.79	1.91 ± 0.36	0.3 ± 0.01**	62.24 ± 5.75	62.67 ± 8.79	58.5 ± 7.15
**A21**	5.55 ± 0.57	1.41 ± 0.11	0.21 ± 0.01	46.8 ± 3.15	37.19 ± 1.91	65.45 ± 8.99
**A25**	**3.89** ± **0.7**	**1.16** ± **0.2**	**0.23** ± **0.02**	**52.13** ± **4.51**	**45.72** ± **4.91**	**57.29** ± **5.77**
**A2**	4.38 ± 0.32	1.38 ± 0.14	0.24 ± 0.01	48.96 ± 5.74	47.32 ± 3.32	57.98 ± 7.37
**A20**	4.55 ± 0.82	1.21 ± 0.19	0.21 ± 0.02	57.09 ± 7.82	33.41 ± 2.53**	52.62 ± 6.64
**A29**	3.14 ± 0.24	0.85 ± 0.06	0.22 ± 0.02	68.81 ± 7.52*	58.72 ± 8.77	54.46 ± 5.34
**A31**	4.3 ± 0.55	1.16 ± 0.16	0.21 ± 0.01	56 ± 8.73	49.83 ± 3.95	50.93 ± 4.56

Statistical significances are based on unpaired *t*-tests (one and two asterisks indicates *P*-values ≤0.1 and 0.05, respectively) and were assessed in comparison to line A25 (bold), which did not show AMY3 suppression. Provided are means ± SE. The WT values are provided as a reference in Figure S6. FW, fresh weight. The harvest index is the storage root FW divided by the sum of the shoot and storage root FWs. See also Dataset S6 for raw data and further statistical analyses.

Sink-source reversal will support energy-consuming processes such as canopy regrowth. Therefore, we tested whether *AMY3A* silencing affected axillary bud sprouting from the remaining stem of pruned plants (see Figure S6 for experimental overview). The number of nodes on the 40-cm stem segment left after pruning was similar between lines (18 to 20; Dataset S6). Despite reduced starch mobilization after pruning in the *AMY3A* RNAi lines, the percentage of nodes sprouting 10 DAP in the AMY3 RNAi lines was largely unchanged relative to the transgenic control and the wild-type (Table 1; Dataset S6). This indicates that there were sufficient sugars in the roots and/or stem to fuel regrowth until the newly emerged leaves became autotrophic. We also assessed the resprouting of stem cuttings (stakes), which is of particular interest since cassava is propagated this way by farmers and because *AMY3A* was suppressed in the stems of the RNAi lines (Fig. 5c). Stakes were replanted in the field, either directly or after storage for 1 wk (Figure S6). In both cases, there were no consistent genotype-specific differences in the resprouting capacity between the AMY3 RNAi lines and the transgenic control plants (Table 1 and Dataset S6). The freshly prepared wild-type stakes produced slightly more biomass, but this was not observed for the stored stakes.

## Discussion

Cassava has many agronomic and physiological qualities rendering it a globally important crop. Its capacity to produce large quantities of starch in its storage roots directly contributes to food security for hundreds of millions of people living in developing economies and makes it an ideal source of income, especially for small-holder farmers. However, remobilization of stored carbohydrates from the storage roots can occur when plants are stressed, which lessens the crop's value and has not been thoroughly investigated so far.

### AMY3A: a key player in storage root starch mobilization

After inducing a sink-source reversal by ratooning the plants, we could correlate the decline in storage root starch content with repression of starch biosynthesis genes and induction of starch degradative genes. Among the starch-degrading factors, AMY3A was strongly and reproducibly induced upon pruning, both at the transcript and protein levels (Fig. 2; Figure S4). This is interesting, since AMY3 orthologs have not yet been shown to have clear roles in starch degradation in crops, and our results therefore suggest a distinct mechanism compared with systems where α-amylases from other classes are known to be important. α-Amylases have been mostly studied in the context of cereal seed starch degradation during germination and seedling establishment (Fincher 1989). This process involves α-amylases belonging to the AMY1 class, secreted from the germinating embryo and the endosperm's aleurone layer into the extracellular space, where it degrades starch in the non-living starchy endosperm. However, *Me*AMY3A is a member of a distinct class that is redox-activated and targeted to plastids (Stanley et al. 2005; Seung et al. 2013), consistent with a role in amyloplast-localized starch degradation within the living cells of the storage root.

The Arabidopsis orthologue of *Me*AMY3A, *At*AMY3, has been studied in detail. It is dispensable for transitory leaf starch degradation under laboratory conditions (Yu et al. 2005). However, when other starch-degrading enzymes are missing, a role for AMY3 becomes evident; it influences starch degradation in the leaf mesophyll (Kötting et al. 2009; Streb et al. 2012; Thalmann et al. 2016) and in stomatal guard cells (Horrer et al. 2016). Under certain circumstances, AMY3 can also degrade glucan primers used for starch granule initiation (Seung et al. 2016) and influence the ability of starch polymers to undergo phase transition into a semi-crystalline state (Streb et al. 2008).

In our transgenic cassava plants with RNAi-mediated suppression of *AMY3A*, the decline of storage root starch levels upon shoot pruning is reduced or prevented (Fig. 6b). This is direct evidence that AMY3A has a role in degrading existing starch granules (although its induction may also suppress the initiation of new granules). That said, it is unlikely that AMY3A works alone in the degradation of storage root starch; its activity will liberate a mixture of linear and branched oligosaccharides from the starch granules, which will need to be metabolized by other enzymes. Indeed, our zymogram analysis revealed a general increase in amylolytic activities and our transcriptomic data point to the induction of other proteins important in starch degradation, which could also represent valuable gene targets for future studies (e.g. β-amylase and β-amylase-like proteins; Fig. 2). Of special note is BAM9, which was recently shown to be a non-enzymatic positive regulator of starch degradation in Arabidopsis (David et al. 2022), and able to interact with and activate AMY3 (Berndsen et al. 2025). MeBAM9 could operate in a similar way to activate AMY3 in cassava roots. Furthermore, it is possible that degradative enzymes could be activated through post-translational mechanisms such as phosphorylation or redox regulation (Kötting et al. 2010; Glaring et al. 2012).

The starch accumulating in the cassava storage root represents a massive carbohydrate resource for the plant. Mobilization of this reserve upon exposure to stresses will provide energy and carbon skeletons supporting the plant's resilience and fueling axillary bud initiation and canopy regrowth. Despite their reduced storage starch degradation upon pruning, the transgenic *AMY3A* RNAi lines were not impaired in their capacity for regrowth, neither from the rootstock nor from stem cuttings, compared with transgenic control plants (Table 1). This is important, since it suggests that the beneficial trait in terms of root quality is not hampered by reduced propagation efficiency. We hypothesize that soluble sugars, together with residual starch degradation, especially in the stem itself (Fig. 1b), could be sufficient to fuel regrowth at this stage.

## Conclusion

Cassava is an increasingly important, climate-resilient crop to produce starch, both for food and industrial application, and AMY3A is a primary candidate for restricting starch mobilization in harvestable cassava storage roots following pruning. We suggest that genes like AMY3A, and others identified here, can be targeted with plant breeding technologies to engineer transgene-free cassava (Bull et al. 2018) with improved storage root qualities.

## Materials and methods

### Plant material, maintenance, and field growth conditions

Cassava (*Manihot esculenta* Crantz) cultivar 60444 stem cuttings were planted in pots containing 70% compost (Klasmann Substrate 2) and 30% Perlite in the glasshouse (14 h photoperiod, 60% relative humidity, 24/17 °C (day/night)). After 2 mo, plantlets had rooted and were transferred to 15-cm round pots containing 40% compost (Klasmann Substrate 2), 10% Perlite, 50% lawn soil (Ricoter), and fertilized with Osmocote (The Scotts Company; 11% N, 11% P, 18% K, 2% MgO). Plants were watered 2 to 3 times per day as required. For RNA-sequencing, proteomics, carbohydrate, and sugar analyses, 5-month-old plants were pruned by cutting the stem 10 cm above soil level.

For field analyses, in vitro plantlets of selected transgenic lines and 60444 WT were propagated in cassava basic medium (1× Murashige & Skoog (MS) medium including Vitamins (Duchefa), 2% [w/v] sucrose and 2 µM CuSO_4_, pH 5.8, 0.3% [w/v] Gelrite) in a controlled environment chamber (Panasonic MLR) at 28 °C, 16-h light/8-h dark cycle. Material was shipped to IITA, Ibadan, Nigeria, and transferred to sterilized soil in plastic bags for acclimatization for approximately 4 wk. Plants were then planted following a random block design in mulched soil in the field (7°29′27.7″N 3°54′13.6″E), which was covered in a net to protect against insect pests and diseases transmitted by them. Data on rainfall, disease scoring, temperature, weeding, and insecticide treatment (imidacloprid and lambda-cyhalothrin) were collated. After 6 mo of growth (May 2018—November 2018), selected plants were pruned by cutting the stem 40 cm above ground. Material transfer and field trials were conducted in accordance with the license agreements of the Federal Ministry of Agriculture and Rural Development (Nigeria), and the Federal Office for Agriculture (Switzerland).

For recovery scoring, two 25-cm cuttings were made using stems from immediately above the 40-cm length of ratooned plants. All stems had a similar number of nodes. Half the stems were directly planted in a randomized block design in the field, while the remainder was stored in plastic bags for 1 wk prior to planting in the field.

### Radiolabeling with ^14^CO_2_

Radiolabeled [^14^C]CO_2_ was supplied to individual cassava leaves using an adaptation of methods described (Kölling et al. 2013). Briefly, a single intact, fully expanded leaf of each of 4 plants was placed inside a custom-made 17.3-liter Plexiglas chamber within an illuminated fume hood. A seal was made around the undamaged petiole using a foam-rubber gasket. A solution of ^14^C-sodium bicarbonate (5.55 MBq; Hartmann Analytic GmbH, Germany; supplied with a specific activity of 2.2 GBq/mmol) was acidified with lactic acid to liberate ^14^CO_2_ within the chamber, which was circulated by an internal fan. Labeling was performed for a 1-h pulse at a light intensity of 150 μmol quanta m^−2^ s^−1^, and temperature of 20 °C, commencing in the middle of the photoperiod. After the pulse, the labeled leaf was removed from the chamber for a 24-h chase period, with the light/dark cycle maintained. Autoradiography was performed as described (Kölling et al. 2013), except that imaging was used a Typhoon scanner (Cytiva).

### Quantification of starch and soluble sugars

Insoluble and soluble carbohydrate extraction was performed as described (Rosado-Souza et al. 2019). In brief, an aliquot of approximately 200 mg from frozen, homogenized material was mixed with ice-cold 0.77 M perchloric acid. Starch and debris were pelleted by centrifugation (18,000 × *g*, 10 min, 4 °C), and the soluble, sugar-containing supernatant was collected and neutralized to pH 7.0 with 2 M KOH, 0.4 M MES. The pellet was resuspended in sterile, distilled water, and the sample centrifuged as before. This process was repeated three times, each time resuspending the pellet in ice-cold, 80% [v/v] ethanol. Soluble sugars (fructose, glucose, sucrose) and starch were quantified using enzyme-coupled spectrophotometric assays.

### Cassava storage root transcriptome analysis using RNA-seq Illumina sequencing

Total RNA was extracted from storage roots at 4 HAP, 1 DAP, 2 DAP, 6 DAP, and 10 DAP (3 biological replicates per time point). As a control, storage roots of unpruned plants were processed at the 0, 4, and 24 h time points (3 biological samples per time point). Frozen root tissues were ground with a 2010 Geno/Grinder (SPEX SamplePrep) at 1300 rpm. Powdered root tissue (90 mg) was extracted with 600 µL 150 mM Tris-boric acid pH 7.5, 2% [w/v] SDS, 50 mM EDTA for 5 min. Potassium acetate (5 M, 66 μL) and absolute ethanol (150 μL) were added. After mixing, 750 µL of chloroform-isoamylalcohol mixture (24:1) was added, and samples were subjected to centrifugation (13,000 × *g*, 3 min, 20 °C. The aqueous phase was collected and mixed at a 1:1 [v/v] ratio with a phenol/chloroform/isoamyl alcohol mixture 25:24:1. After centrifugation (13,000 × *g*, 1 min, 20 °C), 400 µL of the aqueous phase was mixed with 1 mL of absolute ethanol. RNA was precipitated at −80 °C for 30 min and the samples subjected to centrifugation (13,000 × *g*, 30 min, 4 °C). The pellets were washed with 80% [v/v] ethanol and resuspended in DEPC-treated water. After adding LiCl (to a final concentration of 2 M) RNA was precipitated for 16 h at −20 °C. Samples were subjected to centrifugation (13,000 × *g*, 30 min, 4 °C) and the pellet washed with 80% [v/v] ethanol, then air dried and resuspended in 30 μL DEPC-treated water.

RNA was purified using a NucleoSpin RNA Clean-up XS kit (Macherey-Nagel) according to the manufacturer's protocol and following the optional DNAse treatment (rDNase Set). RNA quality was determined with a Qubit v1.0 Fluorometer (Life Technologies) and a Bioanalyzer 2100 System (Agilent Technologies). Samples with a 260 nm/280 nm ratio between 1.8 and 2.1 and a 28S/18S ratio within 1.5 to 2 were processed. One microgram of total RNA was poly-A enriched, and cDNA was synthesized using the TruSeq RNA Sample Prep Kit v2 (Illumina). The qualities and quantities of the enriched libraries were validated using Qubit v1.0 Fluorometer and the Caliper GX LabChip GX (Caliper Life Sciences). The libraries were normalized to 10 nM in 10 mM Tris-HCl pH 8.5, 0.1% [v/v] Tween20. Cluster generation was performed using 10 pM of pooled normalized libraries on the cBOT with a TruSeq PE Cluster Kit v3-cBot-HS (Illumina).

The Illumina HiSeq 2000 System was used to generate the sequence reads, which were analyzed using CLC Genomics Workbench (Qiagen). Low-quality ends of the reads were trimmed (quality score limit of 0.05, maximum of 2 ambiguous nucleotides, 3 bases from the read start, and 10 bases from the read end), and reads shorter than 15 nucleotides were discarded. Reads were mapped to the reference genome (Accession ID LTYI02000000; *Manihot esculenta* v8.1, cultivar AM560-2; Dataset S2). Empirical analysis of differentially expressed genes was performed using multi-group unpaired as parameters (Robinson et al. 2010). RPKM expression values were normalized with the quantile method. The fold change (FC) between all pruned timepoints (4 HAP, 1 DAP, 2 DAP, 6 DAP, and 10 DAP) and their corresponding unpruned controls (4 h unpruned for the first timepoint, 0 and 24 h combined for the latest) was calculated and converted into the Log_2_ scale. To calculate the *P*-value, a Gaussian Statistical analysis was done by doing a *t*-test with homogeneous variance for all pairs.

For cluster and GO term analysis, transcripts were filtered for those showing a log_2_ fold change < (−1) or >1 and a *P*-value <0.001 compared to the normalized gene expression values from the two controls (day 0 and day 1) in at least one condition. The appropriate cluster number was determined by the Elbow method using the “wss” function, followed by transcript grouping into 5 clusters by the “kmeans” function in RStudio (v2024.12.1). Transcripts with a best *Arabidopsis thaliana* blast hit (Best.hit.arabi.defline) were subjected to Fisher's Exact overrepresentation tests in the PANTHER webtool (Thomas et al. 2022) using the corresponding Arabidopsis accessions as input, Bonferroni correction for multiple testing, the database released 2025-07-22 for annotation (DOI: 10.5281/zenodo.16423886) and the Arabidopsis accessions of all detected transcript as background reference.

### Shotgun proteomics

Total proteins were extracted from storage roots (10 to 20 g) prior to pruning (0 DAP) and at 10 DAP (3 biological replicates for each time point) according to a modified protocol (Saravanan and Rose 2004). In brief, root material was homogenized on ice using a glass homogenizer in 1% [w/v] PVPP, 0.7 M sucrose, 0.1 M KCl, 0.5 M Tris-HCl (pH 7.5), 100 mM EDTA, 2% [v/v] β-mercaptoethanol, 2× protease inhibitor cocktail (Roche), mixed in a 1:1 ratio [v/v] with phenol (pH 8; Sigma-Aldrich), and centrifuged for 30 min at 4000 × *g* at 4 °C. Proteins in the phenol phase were precipitated in 5 volumes of 0.1 M ammonium acetate, 100% methanol for 24 h at 20 °C before being centrifuged for 5 min at 4000 × *g* at 4 °C. Samples were washed in 100% methanol and 80% [v/v] acetone. Air-dried precipitated proteins were resuspended in 4% [w/v] SDS, 40 mM Tris pH 6.8, 2× protease inhibitor cocktail (Roche). Protein concentration was determined using a BCA Protein Assay Kit (Thermo Fisher Scientific) before adding 40 mM DTT. Proteins (100 μg) were separated by SDS-PAGE and visualized by staining with Coomassie dye. Each sample lane was fractionated into 14 pieces (Figure S4a) prior to in-gel digestion using trypsin and label-free mass spectrometry as described (Bischof et al. 2011).

Protein abundances were quantified based on precursor signal intensity using Progenesis QI for Proteomics (v4.2.7951.42406; nonlinear dynamics, Waters) as described (Pipitone et al. 2021). The workflow for fractionated samples was used with the following modifications. First, the alignment references were selected automatically among the biological replicates of each fraction. Second, mass spectrometry (MS)/MS spectra were mapped using Mascot Server version (v2.7.0.1) with up to one missed trypsin cleavage allowed, carbamidomethylation of cysteine as fixed modification, N-terminal acetylation and oxidation of methionine as variable modifications, a precursor ion mass tolerance of 10 ppm and a fragment ion tolerance of 0.6 Da, and using a forward and reverse protein database of *Manihot esculenta* v8.1 (Phytozome) including common MS contaminants. Third, the false discovery rate for creating the spectrum reports in Scaffold 6 (v5.1.0, Proteome Software Inc.) was set to 1% and 0.5% at the protein and peptide level, respectively, with at least one peptide per protein. Fourth, proteins were quantified using all peptides associated with the proteins and a minimum of one unique peptide, and all statistics were calculated by Progenesis QI (Dataset S2). As a rule, all samples (3 biological replicates per time point) and peptides were included in the analysis. In total, 3030 cassava proteins (or protein groups) were identified with an estimated false discovery rate of 1.1% at the protein level. One of the 8 detected peptides associated with AMY3A (Manes.05G097100.1.p) was not used for quantification; the MS1 signal ascribed to this peptide (SLHEVGLK) had exceptionally high intensity and lacked multiple ions typically resulting from varying numbers of ^13^C atoms, indicating that it likely derived from a non-peptide contaminant.

For GO term analysis, proteins were filtered for those showing a log_2_ fold change < (−0.585) or >0.585 and a *P*-value <0.05 compared to the control (0 DAP). Proteins with positive or negative log_2_ fold changes were assessed separately. Their corresponding best *A. thaliana* blast hit (Best.hit.arabi.defline), if any, was used as input in Fisher's Exact overrepresentation tests in the PANTHER webtool using Bonferroni correction for multiple testing, the database released 22-07-2025 for annotation (DOI: 10.5281/zenodo.16423886) and the Arabidopsis accessions of all quantified proteins as background reference. Proteins with positive or negative log_2_ fold changes were assessed separately.

### Protein extraction and zymography using native-PAGE

Total soluble proteins were extracted in ice-cold extraction buffer (100 mM MOPS pH 7.2, 1 mM EDTA, 10% [v/v] ethanediol, 1% [w/v] PVPP, 1 mM DTT, 1× cOmplete Protease Inhibitor EDTA-free Mixture (Roche)) and quantified using the Bio-Rad Protein Assay (Bio-Rad). Proteins were loaded in native polyacrylamide gels consisting of a stacking gel (3.75% [w/v]) and a separating gel (6% [w/v]), as described previously (Zeeman et al. 1998b). The separating gels were supplemented with either 0.1% [w/v] solubilized amylopectin from potato starch (Sigma-Aldrich), 0.1 or 0.2% [w/v] β-limit dextrin (Megazyme) or 1% [w/v] Red Pullulan (Megazyme). After electrophoresis, gels were incubated for 2 h at 37 °C in 100 mM Tris pH 7.2, 1 mM MgCl_2_, 1 mM CaCl_2_, 2.5 mM DTT. Amylolytic activity was visualized directly in gels containing red pullulan, or after staining with Lugol solution (Sigma-Aldrich) in gels containing amylopectin or β-limit dextrin.

### Phylogenetic analysis

Cassava AMY genes were identified in the reference genome (Accession ID LTYI02000000; *Manihot esculenta* v8.1, cultivar AM560-2), which provides the best hit in blasting the Arabidopsis genome (Best-hit-arabi-defline). The list of cassava genes described as Arabidopsis AMY1 (AT4G25000), AMY2 (AT1G76130), and AMY3 (AT1G69830) was retrieved. For both organisms, the protein sequences of all identified α-amylases were retrieved from Phytozome (Goodstein et al. 2012). The Arabidopsis and cassava protein sequences were aligned (Dataset S7), and a phylogenetic tree was subsequently constructed using the neighbor-joining method in MEGA v11 (Tamura et al. 2021), using 1000 bootstrap replications. The protein domains were retrieved with the SMART online tool (Letunic et al. 2021).

### Total RNA isolation and RT-qPCR

Total RNA was isolated from 100 mg of storage root using a scaled-down version of a described protocol (Morante-Carriel et al. 2014). Genomic DNA contamination was removed using DNase I (Roche). First-strand cDNA was synthesized from 2 µg RNA using oligo(dT)18 primers and the RevertAid First-Strand cDNA Synthesis Kit (Thermo Fisher Scientific). Two µL of cDNA diluted 5-fold were used in a 10 µL reaction of Fast SYBR Green Master Mix (Thermo Fisher Scientific). Real-time quantitative PCRs were performed using the Light Cycler 480 II system (Roche). The results were standardized using two reference genes selected as the most stable and accurate ones for our conditions: *MeEF1a* and *MeUBQ10* (Moreno et al. 2011). After normalizing the ΔCt to the average value of 0 DAP, the fold change was calculated as being 2^−ΔΔCt^. Efficiencies of primer pairs were measured and confirmed to be similar. Primer sequences are provided in Table S1.

### Confocal microscopy imaging of MeAMY3A-YFP in *Nicotiana benthamiana*

The full-length *MeAMY3A* CDS, including the predicted 50-amino acid chloroplastic transit peptide, was PCR-amplified from storage root cassava cDNA using oligonucleotides designed to remove the stop codon and incorporate attB1 and attB2 sites (Table S1) for subsequent amplicon cloning in pDONR221 (Gateway Technology). The target sequence was then cloned in-frame with the C-terminal YFP reporter sequence and downstream of the CaMV35S promoter in the pK7YWG2 vector (Gateway Technology). The expression vector was used to transform *Agrobacterium tumefaciens* cells, strain GV3101. Cells were incubated overnight at 28 °C, pelleted by centrifugation (5000 × *g*, 15 min, 20 °C), resuspended to an OD_600_ of 0.5 in infiltration medium (50 mM MES pH 5.7, 2 mM NaH_2_PO_4_, 0.5% [w/v] glucose, 100 μM acetosyringone) and incubated at 20 °C for 1 h under gentle shaking. Several *Nicotiana benthamiana* plants were infiltrated manually. Microscopy imaging was performed 3 d post-infiltration using a Zeiss LSM 780 confocal microscope (Carl Zeiss). Signals from YFP and autofluorescence of chlorophyll were recorded using an argon laser at 514 nm wavelength for excitation, and emission was captured between 462 and 500 nm for YFP and 662 and 721 nm for chlorophyll autofluorescence.

### Recombinant protein expression in *Escherichia coli*

The CDS of *MeAMY3A*, lacking the predicted 50-amino acid chloroplast transit peptide at the N-terminus, was amplified by PCR from a cassava storage root cDNA library (primers provided in Table S1), with incorporated BamHI restriction enzyme recognition sites. The amplicon was restriction-digested and ligated into the pProExHTb vector (Invitrogen) to produce *pProExHTb-MeAMY3A*. To obtain the mutated MeAMY3A D675N, a point mutation was introduced in the pProExHTb-MeAMY3A sequence using the QuikChange Site-Directed Mutagenesis Kit (primers provided in Table S1) following the manufacturer's guidelines (Agilent Technologies). The expression vector was transformed into *E. coli* strain BL21 (DE3) CodonPlus cells (Agilent Technologies) and cultured in LB medium at 37 °C until an OD_600_ of 0.5 to 0.6. Protein expression was then induced by addition of IPTG (1 mM final concentration) and incubation for 24 h at 18 °C. Bacterial cells were lysed and the recombinant protein purified using His-TRAP columns as described (Seung et al. 2013).

### α-amylase activity assay

Amylolytic activities of *At*AMY3 and *Me*AMY3A (WT and mutated) were assayed against BPNP-G7 (Ceralpha method assay, Megazyme), using 10 µg of recombinant proteins under reduced state at pH 7.9 for 90 min at 37 °C, as previously described (Seung et al. 2013).

### Design and assembly of pAMY3:RNAi construct and cassava transformation

The nucleotide sequence +1 (ATG) to +210 position of the *AMY3A* (Manes.05G097100.1) coding sequence (Accession ID LTYI02000000; *Manihot esculenta* v8.1, cultivar AM560-2) was used to generate the RNAi construct. PCR-mediated amplification was used to produce the 210 bp amplicon from genomic DNA isolated from cultivar 60444. The amplicon was blunt-end cloned into the vector pJET1.2 following the instructions of the CloneJET PCR Cloning kit (Thermo Fisher Scientific). The cloned AMY3A fragment served as a template for a second PCR amplification using two pairs of oligonucleotides with flanking restriction enzyme recognition sites to later orientate the two amplicons in sense and antisense positions (all primers provided in Table S1). Double-restriction enzyme digestion of the respective fragments was performed and products sub-cloned into a prepared pBluescriptSKII vector, flanking a synthetic plant intron sequence (position 57 to 165 bp, GenBank M27939; Goodall and Filipowicz 1989) to produce the hairpin unit. To generate the final RNAi construct, the hairpin unit was restriction-digested using KpnI and BamHI and ligated into a prepared, modified pCAMBIA1301 vector (Vanderschuren et al. 2014), between the *Solanum tuberosum* class I patatin B33 promoter (GQ352473.1, 11 to 970 bp; Liu et al. 1990; Naumkina et al. 2007) and the NOS PolyA transcription terminator sequence. The T-DNA of pAMY3:RNAi contains the hptII (Hygromycin B) resistance gene expressed by the NOS promoter and CaMV PolyA transcription terminator sequence for selection of hygromycin-resistant (transformed) plant tissue. pAMY3:RNAi was electroporated into *Agrobacterium tumefaciens* strain LBA4404 and used to transform friable embryogenic callus of cassava cultivar 60444, as described (Bull et al. 2009).

### Southern blot analysis to determine T-DNA integration

T-DNA integration numbers were determined by Southern blotting, following a previously established protocol (Bull et al. 2017).

### Total protein extraction and immunoblotting for AMY3

Total soluble proteins were extracted from 100 mg of powdered storage root by homogenizing in 200 µL of 40 mM Tris pH 6.8, 4% [w/v] SDS, and 2× cOmplete, EDTA-free Protease Inhibitor Cocktail (Roche). Twenty µL of extract per sample were separated by SDS-PAGE and the proteins transferred to a Low-Fluorescence PVDF membrane using a Trans-Blot Turbo Transfer System (Bio-Rad). Polyclonal antibodies were raised against the recombinant cassava AMY3A protein (lacking the transit peptide; Eurogentec), which has 84% amino acid identity to AMY3B. Membranes were probed with the *Me*AMY3 antibody and, simultaneously, with an anti-plant-actin antibody (plant; monoclonal/mouse; Sigma-Aldrich clone 10-B3 [MAbGPa]). Proteins were detected using the infrared (IR) fluorescence IR800 and IR700-conjugated secondary antibodies and imaged using the Odyssey CLx Detection System (LI-COR Biosciences).

### Research data

Research data presented in this work are available in Datasets S1 to S7. RNA-seq and data can be found in the NCBI GEO database. Mass spectrometry proteomics data have been deposited to the ProteomeXchange Consortium via the PRIDE partner repository (Perez-Riverol et al. 2022) with the dataset identifier PXD045690.

### Accession numbers

Relevant sequence information related to this article can be found under the gene codes mentioned in Fig. 4, Figure S5, and Dataset S2.

## Supplementary Material

kiag341_Supplementary_Data

## Data Availability

The data underlying this article are available in the article and in its online supplementary material. Proteomics data are available on the PRIDE database (project code PXD045690).
