# Supplementary material for: Understanding and modifying starch metabolism to limit yield losses in field-grown cassava
Source: Plant Physiol. 2026 Jun 4;201(3):kiag341. doi: 10.1093/plphys/kiag341 (PMC13368610; doi:10.1093/plphys/kiag341)
Supplement: kiag341_Supplementary_Data [file kiag341_supplementary_data.zip › David_et_al_Supplementary_Material_Revised_AmericanEnglish.pdf]

UNDERSTANDING AND MODIFYING STARCH METABOLISM TO LIMIT YIELD LOSSES IN FIELD-GROWN CASSAVA

Laure C. David, Gabriel Deslandes-Hérold, Carmen Hostettler, Sylvain Bischof, Michaela Fischer-Stettler, Anna V. Carluccio, Barbara Pfister, Gavin M. George, Wuyan Wang, Livia Stavolone, Andreas Gisel, Simon E. Bull, Melanie R. Abt, and Samuel C. Zeeman

SUPPLEMENTARY FIGURES AND TABLES

Supplementary Figs. S1-S6
Supplementary Table S1

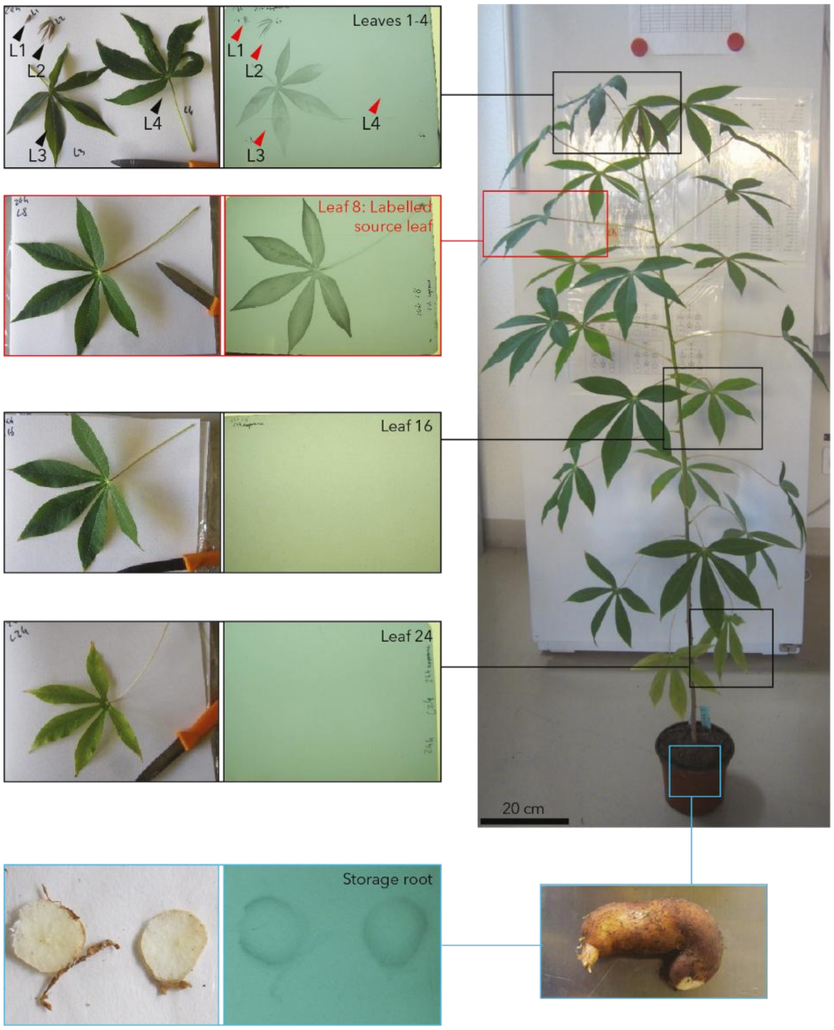

Supplementary Figure S1. $^{14}CO_2$ labelling of leaf 8 to track export of photo-assimilates. Under illumination, leaf 8 was exposed to a 1-h pulse of $^{14}CO_2$. After a 24-h chase period, the leaves and storage roots were collected and analyzed by autoradiography to detect assimilate export. Sink tissues (including leaves 1 to 3 and the outer ring of storage root sections) contained exported radioactivity, while unlabeled source tissues (leaves 4, 16 and 24) did not. Images from one of 4 labelled plants are shown.

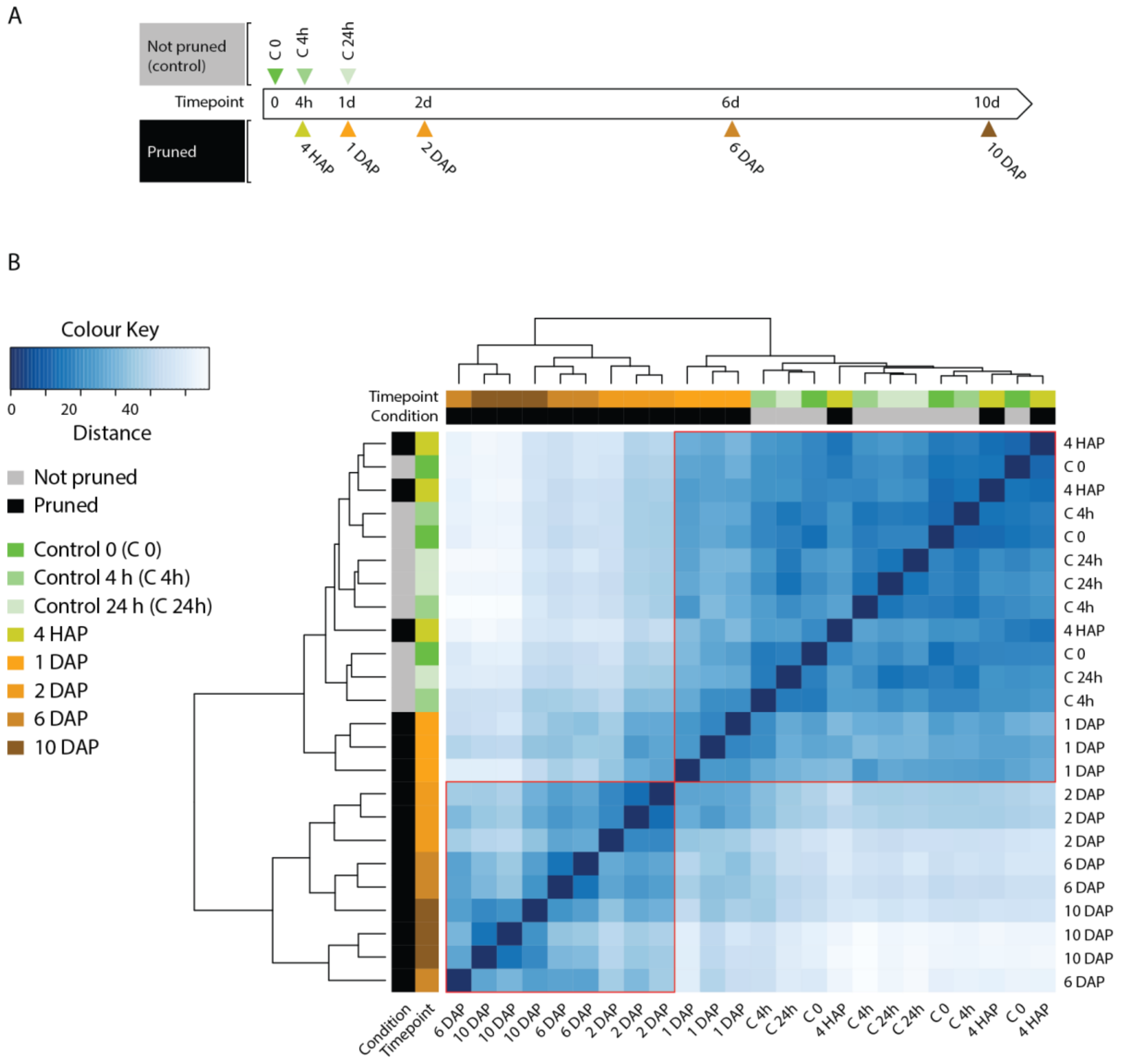

Supplementary Figure S2. Sampling strategy and assessment of RNAseq experiment. **A)** Summary of the sampling protocol. Storage root samples were taken from 5-month-old plants at different time points (arrows). h, hours; HAP, hours after pruning; d, day; DAP, days after pruning. **B)** Clustering sample-to-sample distance. Heatmap (based on 10,775 transcripts, each of which had more than 100 reads mapped) of the Pearson correlation between the expression levels of differentially expressed genes. Dark blue color marks highly correlated samples, while light blue color marks low correlation. The two main clusters are marked by red boxes. The first cluster contains all controls and pruned samples up to 1 DAP (upper right). The second cluster contains samples from 2 to 10 DAP (lower left). See Supplementary Dataset S2.

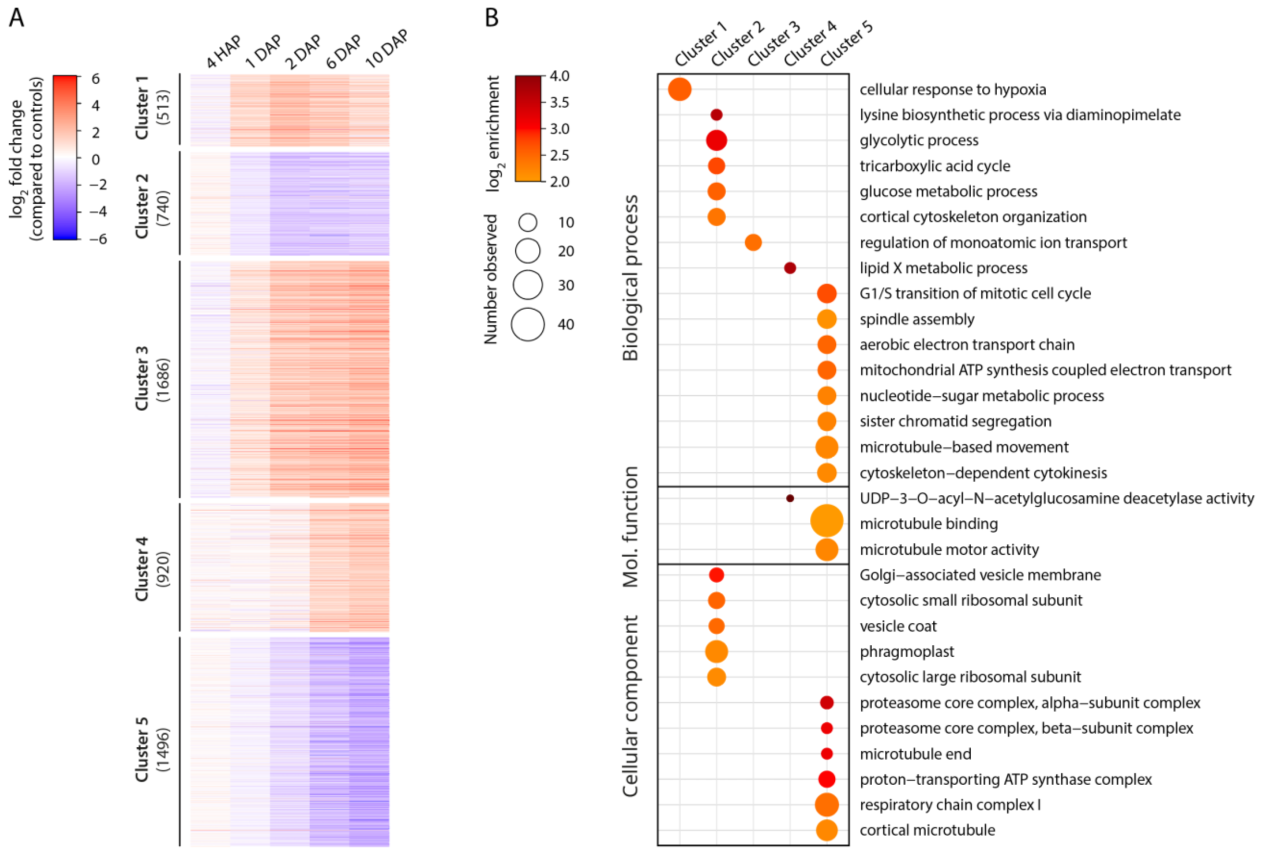

Supplementary Figure S3. Cluster and gene ontology (GO) term analysis of differentially expressed genes. Log_2 fold changes of transcripts from storage roots 4 h after pruning (HAP) or 1, 2, 6, 10 days after pruning (DAP) were calculated compared to the normalized gene RNA-sequencing gene expression values from storage roots of unpruned plants harvested on two consecutive days (day 0 and day 1). **A)** Heatmap of log_2 fold changes of transcripts showing significant changes (log_2 FC <|1|, p-value <0.001) compared to the controls in at least one group. Clusters were determined by k-means clustering. The number of transcripts in the clusters are given in brackets. **B)** GO terms enriched in the individual transcript clusters. The best Arabidopsis blast hits were used as input in PANTHER overrepresentation tests. GO terms were filtered for log_2 enrichment ≥2 and an adjusted p-value <0.05. Only the most specific subclass within a GO term family is shown. Circle size indicates the observed number of transcripts of a GO term. Mol. function, molecular function. For underlying data see Supplementary Dataset S2.

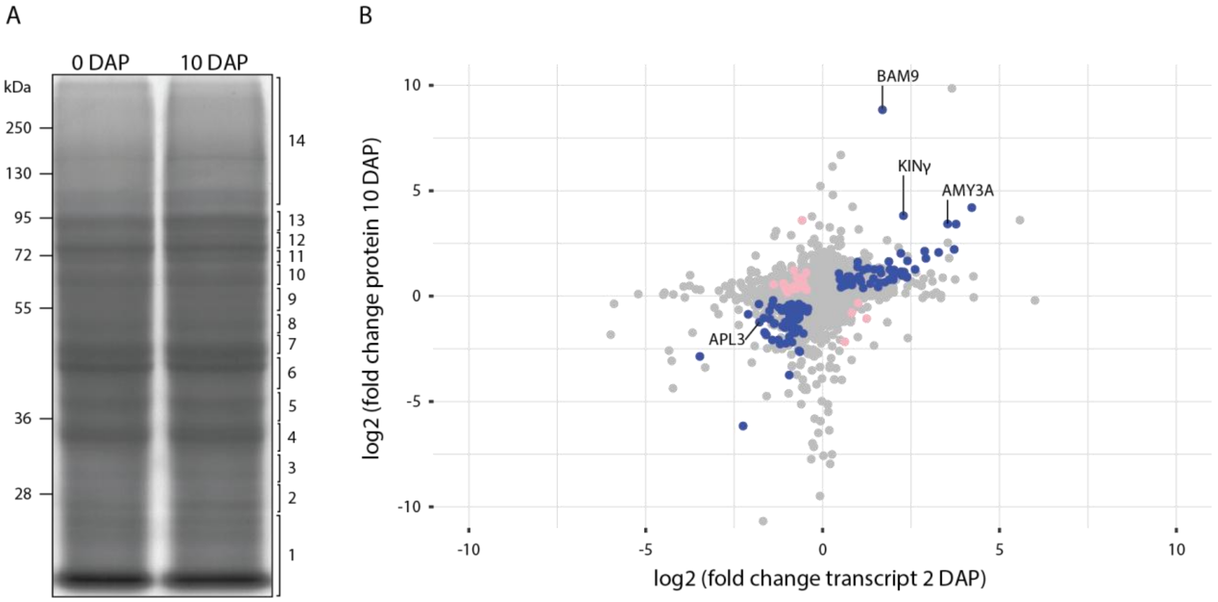

Supplementary Figure S4. Comparison of the proteomic and transcriptomic experiments. **A)** Representative pictures of storage root protein extracts fractionated by SDS-PAGE and Coomassie stained. Equal amounts of proteins (100 µg) were loaded for the 0 and 10 days after pruning (DAP) samples. Six unique peptides of AMY3A were identified in fraction 12. **B)** Scatter plot of log_2 fold-change values of transcript/protein pairs. Colored dots represent the 148 transcript/protein pairs exhibiting significantly different expression, i.e. a change in transcript abundance (p-values $\leq$ 0.001) and a change in protein abundance (p-values $\leq$ 0.05). Of these, 120 genes behaved similarly, with the transcript and the corresponding protein changing in the same way (dark blue points), while 28 behaved divergently with the transcript and the corresponding protein changing in the opposite way (pink points). The transcript/protein pairs with no significant difference are displayed in grey. See Supplementary Dataset S2.

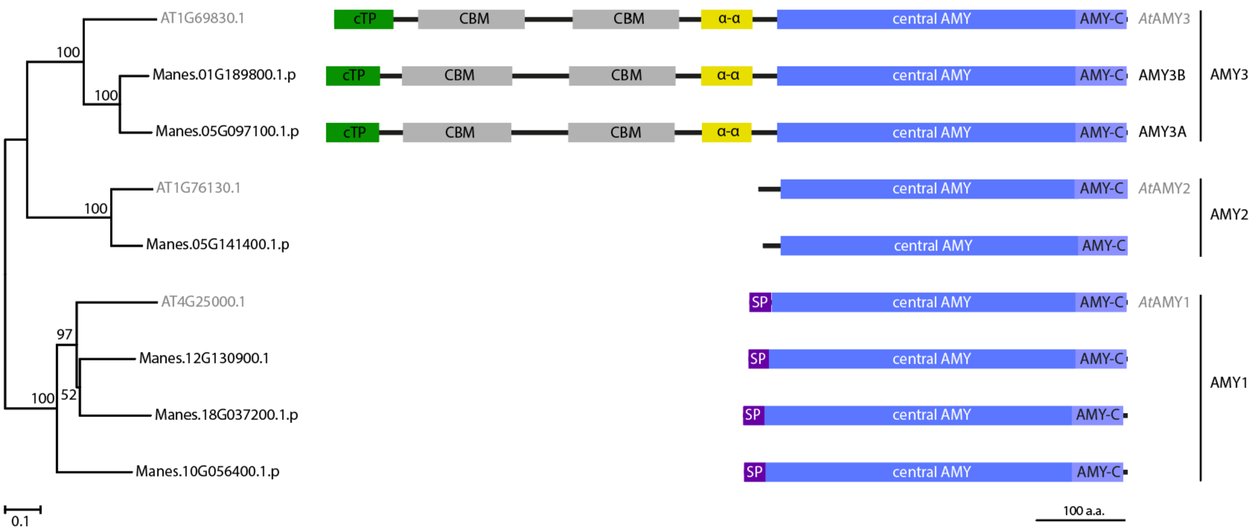

Supplementary Fig. S5. Analysis of the α-amylase family in Arabidopsis and cassava. Phylogenetic tree of the AMY protein family in Arabidopsis and cassava cultivar AM560-2 and domain comparison. The evolutionary history was inferred using the Neighbor-Joining method. Bootstrap values (1000 replicates) over 50 are indicated at branch points. The evolutionary distances were computed using the Poisson correction method and are in the unit of the number of amino acid substitutions per site. InterPro (Paysan-Lafosse et al., 2022) was used to define the carbohydrate-binding modules of family 45 (CBM), the central, catalytic α-amylase domains (central AMY) and the C-terminal α-amylase domains (AMY-C). The αlpha-alpha hairpin structures (α-α) previously identified by Berndsen et al. (2025) were annotated based on protein sequence alignments and AlphaFold3 (Abramson et al., 2024) structural predictions. Chloroplast transit peptides (cTPs) and signal peptides (SP) were predicted by TargetP 2.0 (Armenteros et al., 2019). Arabidopsis proteins are in gray. a.a., amino acids; At, *Arabidopsis thaliana* (reference genome TAIR10); Manes, *Manihot esculenta* AM560-2 (reference genome *Manihot esculenta* v8.1; Accession ID LTYI02000000).

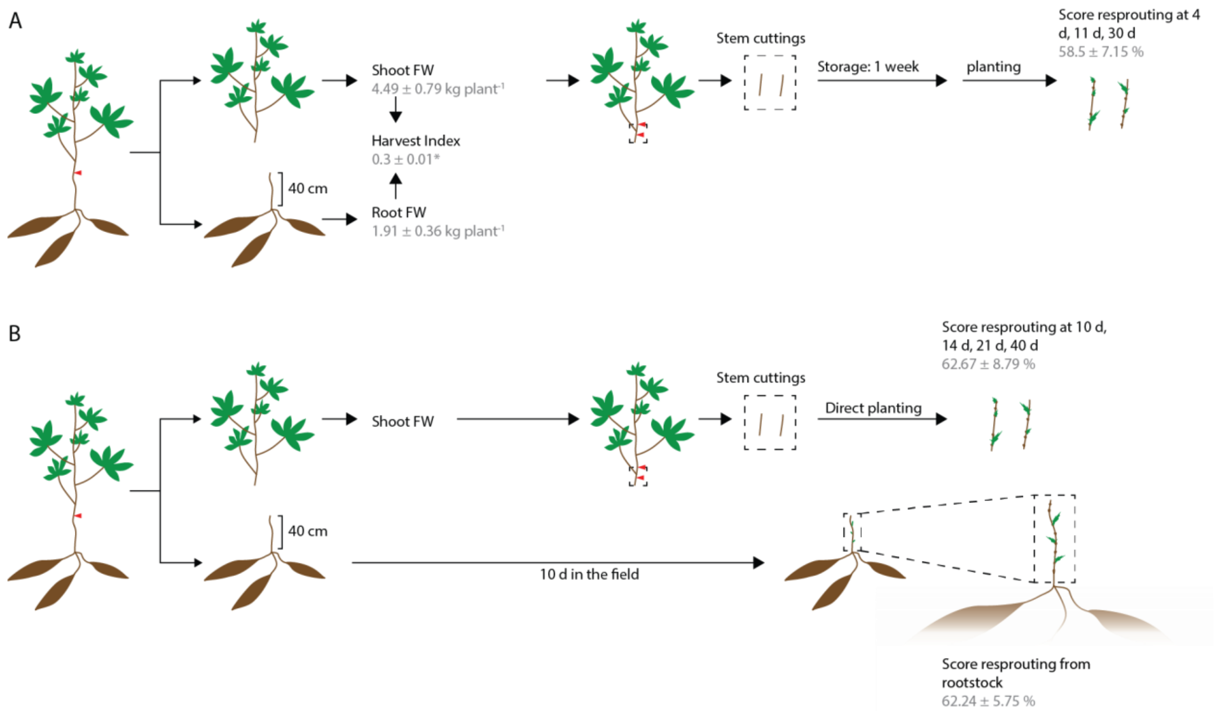

Supplementary Figure S6. Experimental setup used to assess agronomic parameters of field-grown AMY3 RNAi lines grown in a protected field site in Nigeria. **A)** Agronomic properties were assessed after normal growth and **B)** post-pruning, as indicated. Parameters summarized in Table 1 are highlighted by bold font; as reference, the respective wild-type values are provided in grey. See Table 1 and Supplementary Dataset S6A-D.

Supplementary Table S1. Oligonucleotide primers used in this study.

RT-qPCR	Forward primer 5'-3'	Reverse primer 5'-3'
**MeAMY3A**	AAACCGGAAAAAGGTCCAGT	CACAGCCAAAACCCATCTTT
**MeAMY3B**	CATATGGTGAAACGGATCACAG	CCACCATCCCATAACTCCAG
**MeEF1a**	TGAACCACCCTGGTCAGATTGGAA	AACTTGGGCTCCTTCTCAAGCTCT
**MeUBQ10**	TGCATCTCGTTCTCCGATTG	GCGAAGATCAGTCGTTGTTGG

Cloning	Forward primer 5'-3'	Reverse primer 5'-3'
**MeAMY3A-cTP in pK7YWG2**	GGGGACAAGTTTGTACAAAAAAGCAGGCT TCACCATGTCCACCATTTCCATTGAG	GGGGACCACTTTGTACAAGAAAGCTGGGTC AGATGCTTCCCAGACCTTGTA
**MeAMY3A-cTP in pProExHTb**	ATGCGGATCCCCCCATCGGGCCTATACAG	ATGCGGATCCTTAAGATGCTTCCCAGACCT TG
**MeAMY3A-cTP D675N in pProExHTb**	GATGGATGGAGGCTTAATTTTGTCAGAGG ATT	AATCCTCTGACAAAATTAAGCCTCCATCCA TC
**AMY3 RNAi construct: 210 bp amplicon from genomic DNA**	ATGTCGACCGTTGCCATTGAG	AAAAGTTTCAAGAAGAGCGGT
**AMY3 RNAi construct: AMY3 sense orientation, with XhoI/BamHI sites**	ATCTCGAGATGTCGACCGTTGCCATTGAG	ATGGATCCAAAAGTTTCAAGAAGAGCGG
**AMY3 RNAi construct: AMY3 antisense orientation, with ClaI/KpnI sites**	ATATCGATAATCTTACCTCACGAGTGGTA CATGTCGACCGTTGCCATTGAG	ATGGTACCAAAAGTTTCAAGAAGAGCGGT

Supplementary References

J. Abramson *et al.* (2024) Accurate structure prediction of biomolecular interactions with AlphaFold 3. Nature **630:** 493–500

Armenteros JJA, Salvatore M, Emanuelsson O, Winther O, Von Heijne G, Elofsson A, Nielsen H (2019) Detecting sequence signals in targeting peptides using deep learning. Life Science Alliance **2:** 1-15

Emanuelsson O, Nielsen H, Heijne GV (1999) ChloroP, a neural network-based method for predicting chloroplast transit peptides and their cleavage sites. Protein Science **8:** 978–984

T. Paysan-Lafosse *et al.* (2023) InterPro in 2022. Nucleic Acids Research **51:** D418–D427
